# Respiratory management of critically ill pneumocystis pneumonia patients: a multicenter retrospective study

**DOI:** 10.1186/s13613-025-01503-6

**Published:** 2025-08-06

**Authors:** Florian Reizine, Vicky Stiegler, Romain Lécuyer, Benoit Tessoulin, Marie Gallais, Fabrice Camou, Florent Morio, Anne Cady, Frederic Gabriel, Emmanuel Canet, François Raffi, David Boutoille, Nahéma Issa, Benjamin Gaborit, the PRONOCYSTIS Study Group, Charlotte Biron, Maeva Lefebvre, Benjamin Jean Gaborit, Paul Le Turnier, Colin Deschanvres, Raphael Lecomte, Marie Chauveau, Antoine Asquier-Khati, Valentin Pineau, Marie Prime, Clarisse Delaunay, Hakim Essid, Vicky Steigler, Patrice Le Pape, Rose-Anne Lavergne, Fakhri Jeddi, Stéphane Corvec, Pascale Bemer, Jocelyne Caillon, Aurélie Guillouzouic, Anne-Gaëlle Leroy, Karim Lakhal, Raphaël Cinotti, Antoine Roquilly, Mickael Vourc’h, Jean Reignier, Soraya Benguerfi, François Xavier Blanc, Cédric Bretonniere, Jean Morin, Camille Le Blanc, Hakim Alami, Olivier Guisset, Gaelle Mourissoux, Isabelle Accoceberry, Noémie Coron, Laurence Delhaes, Sébastien Imbert, Maxime Lefranc, Florian Lussac-Sorton, Amandine Rougeron, Marie Gousseff, Yoann Crabol, Grégory Corvaisier, Florent Lautredoux, Myriam Auger, Pascal Pouedras

**Affiliations:** 1 Service de Réanimation Polyvalente, Centre Hospitalier Bretagne-Atlantique, 56000 Vannes, France; 2 Department of Infectious Diseases, INSERM, University Hospital of Nantes and Centre d’Investigation Clinique 1413, Nantes, France; 3 UR 1155, Laboratory of Targets and Drugs for Infections and Cancer, IRS2-Nantes Biotech, Nantes, France; 4 Infectious Diseases Department, Centre Hospitalier de Cholet, Cholet, France; 5 INSERM, U1232, Hematology Department, Nantes University Hospital, CRCI2NA, Nantes University, Nantes, France; 6 Internal Medicine and Infectious Disease Unit, Groupe Sud, University Hospital, Bordeaux, France; 7 Cibles et Médicaments des Infections et de l’Immunité, Nantes Université, CHU Nantes, Nantes, France; 8 Department of Microbiology, Centre Hospitalier Bretagne-Atlantique, Vannes, France; 9 Service de Parasitologie Mycologie, Centre Hospitalier Universitaire de Bordeaux, 33000 Bordeaux, France; 10 Medical Intensive Care, University Hospital, Nantes, France; 11 EA 3826, Laboratory of Clinical and Experimental Therapeutics of Infections, IRS2-Nantes Biotech, Nantes, France; 12 Infectious Diseases Department, Hôtel-Dieu University Hospital, 1 Place Alexis-Ricordeau, 44000 Nantes, France

**Keywords:** *Pneumocystis jirovecii* pneumonia, Acute respiratory failure, Intensive care unit, High-Flow Nasal Cannula, Standard oxygen, Respiratory management

## Abstract

**Background:**

*Pneumocystis jirovecii* pneumonia (PjP) is a rising cause of acute respiratory failure in immunocompromised patients, often requiring Intensive Care Unit (ICU) admission. However, optimal ventilatory strategies remain unclear.

**Methods:**

For the present study, we conducted an ancillary analysis of the PRONOCYSTIS study, a large multicenter cohort of PjP patients. Patients admitted to the ICUs were compared according to initial respiratory management (High-Flow Nasal Cannula (HFNC), standard Oxygen (SO) or Non-Invasive Ventilation (NIV). A propensity score adjustment [inverse probability of treatment weighting (IPTW) analysis] was implemented to account for potential confounders. The primary outcome was intubation rate. Univariable and multivariable Cox regressions were also used to assess variables associated with survival.

**Results:**

Over the study period, 248 patients with PjP were included in the present analysis. Of those, 70 were treated by HFNC while 118 and 60 received SO and NIV, respectively. HFNC patients had a decreased intubation rate (28.6% versus 45.0% in NIV and 55.4% in SO patients; p = 0.003). When assessing the impact of respiratory management on intubation by IPTW, HFNC remained an independent protective factor (weighted Hazard Ratio (HR) 0.41 (95% CI 0.24–0.69); p < 0.001). While, NIV was not associated with intubation (HR 0.62 (95% CI 0.37–1.02); p = 0.056). Through adjusted survival analysis, long-term corticosteroids treatment (aHR 4.03 (95% CI 2.01–8.08); p < 0.001), Solid tumor (aHR 3.37 (95% CI 1.45–7.86); p = 0.005) and the Sequential Organ Failure Assessment score (aHR 1.24 (95% CI 1.15–1.35); p < 0.001) were found to be independent predictor for death. Initial respiratory support was not associated with survival either in the Cox multivariable analysis or in the IPTW analysis.

**Conclusion:**

Through this multicenter observational study of severe PjP patients, although oxygenation strategy was not associated with D90 survival, HFNC support appeared to be associated with a lower intubation rate. Further prospective studies are warranted to refine respiratory management in critically ill PjP patients.

**Supplementary Information:**

The online version contains supplementary material available at 10.1186/s13613-025-01503-6.

## Introduction

Pneumocystis pneumonia (PjP) is a common fungal opportunistic infection in immunocompromised ICU patients, caused by *pneumocystis jirovecii* [1]. The advent of antiretroviral in patients with human immunodeficiency virus (HIV), along with the widespread use of immunosuppressive drugs for hematopoietic stem cell or solid organ transplantation (SOT), hematological malignancies (HM), and immune-mediated inflammatory disease (IMID), has led to a paradigm shift [2]. This has resulted in a rising incidence of PjP with distinct clinical features and a more severe prognosis in HIV-negative patients [3–6]. Nonetheless, acute respiratory failure (ARF) remains the most common clinical presentation of PjP, often necessitating ICU admission in severe cases [3, 4]. Moreover, in HIV-negative patients, PjP is an independent risk factor for requiring mechanical ventilation during ARF in ICUs [7]. During the last decades, the management landscape of ARF has evolved with High-flow Nasal Cannula (HFNC) emerging as a frequently used noninvasive respiratory support in acute settings [8]. While its impact on patients’ survival remains debated [9, 10], certain conditions may benefit more from HFNC. Among them, PjP patients, given their vulnerability, could experience a significant survival advantage through reduced intubation rates [6]. Therefore, we aimed to assess the impact of respiratory management of critically ill PjP patients.

## Method

### Study design

We conducted an ancillary analysis of the PRONOCYSTIS study [11], a large retrospective multicenter cohort study conducted in three French hospitals from January 2011 to December 2021, including patients with a mycological diagnosis of probable or proven PjP according to the European Organization for Research and Treatment of Cancer and the Mycoses Study Group definition [12]. The study protocol was approved by the local ethics committee (Groupe Nantais d’Éthique dans le Domaine de la Santé; Ref. 20,200,217). Therefore, In the present analysis, only patients with probable or proven PcP as defined by the EORTC were included [13].

### Study population and definitions

For the present analysis, patients that were not admitted to the ICU as well as those having missing variable data needed for analysis (regarding respiratory support, need for intubation, and survival) were excluded. Patients were assessed retrospectively and followed up from hospital admission (D0) and until Day-90 after hospital admission. Patients were classified into three groups according to the use of HFNC (HFNC group), of standard oxygen support (SO group) or of Non-Invasive Ventilation (NIV group) for the management of ARF without minimum duration of ventilatory support. Patients receiving NIV with SO or NIV with HFNC between NIV sessions were classified in the NIV group.

The primary objective was to evaluate the intubation rate according to initial respiratory management. Secondary objectives were to compare initial characteristics and clinical courses (including whether patients required intubation) according to initial respiratory management. Finally, we sought to determine variables associated with survival.

Collected data included age, gender, obesity, chronic kidney disease, chronic lung disease, long-term corticosteroid use (defined as > 7 days at a dose ≥ 1 mg/kg/day (prednisone equivalent) or for > 3 months at a lower dose), underlying cause of immunosuppression (classified as SOT, HM, solid tumor and IMID) along with anti-*pneumocystis* prophylaxis use. Initial laboratory results and management variables recorded included diagnosis criteria of PjP (proven or probable), C-reactive protein (CRP). Arterial oxygen tension (PaO_2_) to inspiratory oxygen fraction (FIO_2_) ratio in patients treated with standard oxygen was assessed by using the 4% formula (FiO_2_ = 0.21 + 4% per supplemental liter of oxygen) [14]. Severity was assessed by the Simplified Acute Physiological Score II (SAPS II) and the Sequential Organ Failure Assessment (SOFA) score at ICU admission [15, 16]. Acute Respiratory Distress Syndrome (ARDS) and its degree of severity have been classified according to the most recent definitions, whether patients were on mechanical ventilation or not [17].

### Statistical analysis

Continuous variables were reported as median (interquartile range (IQR)) and were compared between groups using the Kruskall-Wallis test. Categorical variables were displayed as frequency (percentages) and compared using the Fisher’s or the Chi-squared tests, as appropriate. Survival analysis was conducted using univariable and multivariable Cox regression models. Since the initiation of ventilatory support can vary over time, ventilatory support (SO, NIV or HFNC) was modeled as a time-dependent covariate in Cox models to appropriately account for the timing of initiation of respiratory supports and its potential impact on survival. For patients with sequential SO then NIV or SO then HFNC, time to event was considered after the initiation of NIV or of HFNC. Variables that yielded p-values smaller than 0.20 by univariate analysis were considered for the multivariable models. To adjust for baseline covariates that could potentially confound the association between respiratory support and intubation, a propensity score (PS) was estimated for each participant. The PS model was fit using multinomial logistic regression with respiratory support as the dependent variable and the following covariates as independent variables (these variables were selected as they yield a p-value < 0.20 in univariate analyses of both respiratory support and intubation): Age, sex, SOT, chronic pulmonary disease, CRP, PaO_2_ to F_i_O_2_ ratio, SAPS II and SOFA scores. Specifically, we used the multinom function from the R package nnet to fit the multinomial logistic regression model. Stabilized weights were calculated as the marginal probability of receiving the observed treatment divided by the individual predicted probability from the multinomial model. Extreme weights were truncated at the 1st and 99th percentiles to reduce the influence of outliers and improve balance. Covariate balance before and after weighting was assessed using standardized mean differences (SMDs), with values below 0.15 considered indicative of adequate balance. The primary outcome was the incidence of endotracheal intubation. Time-to-event analysis was performed using weighted Cox proportional hazards models, with time zero defined as the start of ventilatory support. The event was defined as intubation, and patients were censored at hospital discharge or death without intubation. The hazard ratios (HRs) and 95% confidence intervals (CIs) for each ventilatory mode were estimated using robust standard errors to account for the use of weights. A second IPTW analysis was conducted to assess the effect of respiratory strategies on survival. The second PS model was built using variables associated with respiratory support and survival. Finally, we also carried out a survival analysis including mechanical ventilation among covariates assessed (and without the type of initial respiratory support).

Using the “MICE” R package and assuming that missing data were randomly missing, multiple imputation using chained equations was used to handle baseline missing values (obesity, chronic renal disease, chronic pulmonary disease, CRP, PaO_2_ to F_i_O_2_ ratio, PaCO_2_, SAPS II and SOFA score). All statistical analyses were two-sided, and p-values less than 0.05 were considered statistically significant. Statistical analyses were done using R software version 4.2.3 (https://www.rproject.org).

## Results

### General characteristics

**Fig. 1:**
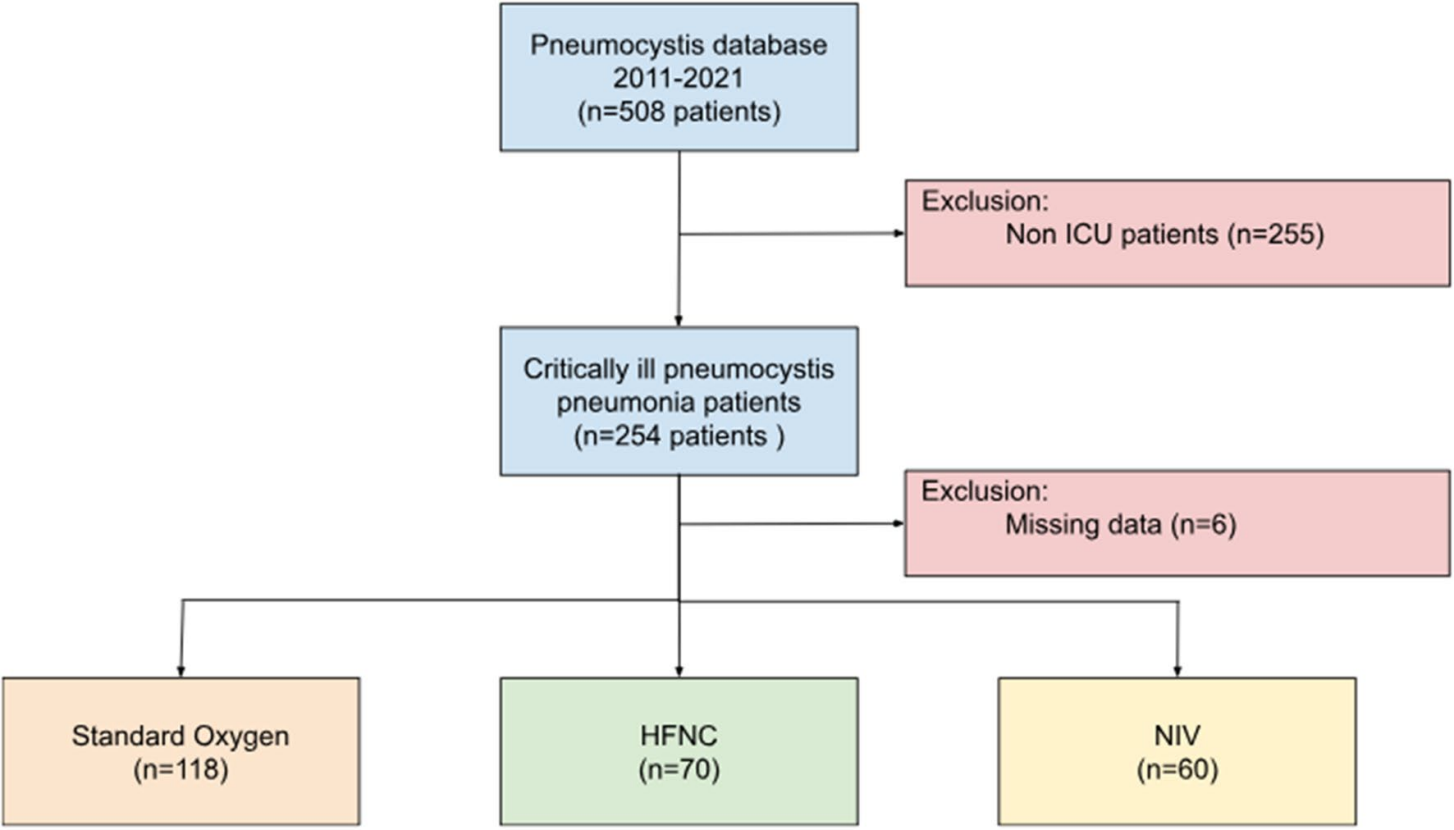
Flow chart of the study population

Over the study period, 248 patients were included in the present analysis. Of those, 70 28.2%) were treated by HFNC while 118 (47.6%) and 60 (24.2%) received SO and NIV, respectively (Fig. 1). Of the 60 patients receiving NIV, 33 (55%) were treated with HFNC between NIV sessions. Baseline characteristics of these patients according to initial respiratory support are presented in Table 1. NIV patients were more frequently male (76.7% versus 58.6% in HFNC patients and 57.6% in SO patients; p = 0.033) and had more commonly a SOT as underlying cause of immunosuppression (35% versus 18.6% in HFNC and SO patients; p = 0.030). Otherwise, there was no difference in baseline characteristics between patient groups. Median SAPS II was 38 (IQR 30–47) while 111 patients (44.8%) required mechanical ventilation (MV). The global mortality rate at Day- 90 was 34.7% (86/248) and reached 55.0% in patients requiring MV (versus 18.2% in non-intubated patients; p < 0.001) **(**Table 2**).** Median time between ICU admission and intubation was 2 (1–4) days.

**Table 1:** Baseline characteristics and outcomes of critically ill pneumocystis pneumonia patients according to respiratory management (N = 248)

	Overall patients n = 248	Standard oxygen n = 118	HFNC n = 70	NIV n = 60	*p*-value
Baseline features					
Age (years)	60 [48–70]	59 [49–70]	63 [46–72]	60 [50–66]	0.679
Male sex	155 (62.5)	68 (57.6)	41 (58.6)	46 (76.7)	0.033
Comorbidities					
Obesity (BMI > 30)^a^	16 (8.3)	7 (7.4)	7 (13.2)	2 (4.4)	0.268
Chronic renal disease^b^	98 (39.8)	45 (38.8)	29 (41.4)	24 (40.0)	0.938
Chronic pulmonary disease^c^	55 (22.4)	28 (24.1)	17 (24.3)	10 (16.7)	0.477
Long term corticosteroids treatment	130 (52.4)	57 (48.7)	36 (51.4)	37 (61.7)	0.256
Underlying cause of immunosuppression					
HIV	57 (23.0)	26 (22.0)	17 (24.3)	14 (23.3)	0.936
Solid organ transplantation	56 (22.4)	22 (18.6)	13 (18.6)	21 (35.0)	0.030
Hematological malignancy	58 (23.4)	32 (27.1)	18 (25.7)	8 (13.3)	0.105
Solid tumor	21 (8.5)	13 (11.0)	6 (8.6)	2 (3.3)	0.220
Immune-mediated inflammatory disease	54 (21.8)	24 (20.3)	15 (21.4)	15 (25.0)	0.773
Initial laboratory findings and management					
Anti pneumocystis prophylaxis	28 (11.3)	15 (12.7)	8 (11.4)	5 (8.3)	0.683
CRP (mg/L)^d^	117 [56–189]	95 [49–181]	131 [60–198]	124 [67–188]	0.316
PaO_2_ to F_i_O_2_ ratio^e^	135 [90–209]	135 [92–215]	122 [90–177]	155 [94–220]	0.248
PaCO_2_^f^	33 [29–37]	33 [29–38]	33 [29–35]	33 [28–39]	0.408
SAPS II^g^	38 [30–47]	39 [29–49]	37 [30–41]	39 [31–50]	0.195
SOFA^h^	4 [3–6]	4 [3–7]	3 [3–5]	4 [3–5]	0.333
Clinical course and outcomes					
90-Day mortality rate	86 (34.7)	51 (43.2)	18 (25.7)	17 (28.3)	0.025
Mechanical ventilation	111 (44.8)	64 (54.2)	20 (28.6)	27 (45.0)	0.003
ARDS^i^	207 (92.4)	90 (87.4)	62 (95.4)	55 (98.2)	0.027
ARDS severity^i^					0.137
Mild ARDS	49 (21.9)	23 (22.8)	8 (12.5)	18 (30.5)	
Moderate ARDS	100 (44.6)	46 (45.5)	29 (45.3)	25 (42.4)	
Severe ARDS	75 (33.5)	32 (31.7)	27 (42.2)	16 (27.1)	
Hospital length of stay	23 [16–36]	22 [14–36]	24 [17–30]	27 [18–44]	0.061
ICU length of stay	8 [5–16]	7 [3–14]	8 [5–15]	13 [6–25]	0.001

Data are presented as median (IQR: interquartiles), n (%)

*BMI* Body mass index, *CRP* C-reactive Protein, *HFNC* High Flow Nasal Cannula, *HIV* Human Immunodeficiency Virus, *ICU* Intensive care Unit, *NIV* Non-invasive ventilation, *SAPS II* Simplified Acute Physiology Score II, *SOFA* Sequential Organ Failure Assessment

^a^Missing data: n = 56; ^b^Missing data: n = 2; ^c^Missing data: n = 2; ^d^Missing data: n = 42; ^d^Missing data: n = 7, ^f^Missing data: n = 39; ^g^Missing data: n = 13, ^h^Missing data: n = 17; ^I h^Missing data: n = 24

**Table 2:** Baseline characteristics and outcomes of critically ill pneumocystis pneumonia patients according to mechanical ventilation (N = 248)

	Overall patients n = 248	No Mechanical ventilation n = 137	Mechanical ventilation n = 111	*p*-value
Baseline features				
Age (years)	60 [48–70]	60 [46–71]	59 [51–68]	0.711
Male sex	155 (62.5)	81 (59.1)	74 (66.7)	0.277
Comorbidities				
Obesity (BMI > 30)^a^	16 (8.3)	11 (10.3)	5 (5.9)	0.405
Chronic renal disease^b^	98 (39.8)	51 (37.2)	47 (43.1)	0.420
Chronic pulmonary disease^c^	55 (22.4)	35 (25.7)	20 (18.2)	0.208
Long term corticosteroids treatment	130 (52.4)	70 (51.1)	60 (54.5)	0.681
Underlying cause of immunosuppression				
HIV	57 (23.0)	33 (24.1)	24 (21.6)	0.759
Solid organ transplantation	56 (22.4)	31 (22.6)	25 (22.5)	1.000
Hematological malignancy	58 (23.4)	35 (25.5)	23 (20.7)	0.458
Solid tumor	21 (8.5)	14 (10.2)	7 (6.3)	0.384
Immune-mediated inflammatory disease	54 (21.8)	25 (18.2)	29 (26.1)	0.180
Initial laboratory findings and management				
Anti pneumocystis prophylaxis	28 (11.3)	16 (11.7)	12 (10.8)	0.990
CRP (mg/L)^d^	117 [56–189]	102 [54–178]	135 [67–211]	0.034
PaO_2_ to F_i_O_2_ ratio^e^	135 [90–209]	180 [115–240]	100 [78–143]	< 0.001
PaCO_2_	33 [29–37]	33 [30–37]	33 [28–38]	0.960
SAPS II^f^	38 [30–47]	36 [28–41]	42 [36–57]	< 0.001
SOFA score^g^	4 [3–6]	3 [2–5]	5 [4–8]	< 0.001
Clinical course and outcomes				
90-Day mortality rate	86 (34.7)	25 (18.2)	61 (55.0)	< 0.001
ARDS^h^	207 (92.4)	110 (88.7)	97 (97.0)	0.038
Hospital length of stay	23 [16–36]	22 [16–31]	26 [15–43]	0.031
ICU length of stay	8 [5–16]	6 [3–9]	15 [8–24]	< 0.001

Data are presented as median (IQR: interquartiles), n (%)

*ARDS* Acute Respiratory Distress Syndrom, *BMI* Body mass index, *CRP* C-reactive Protein, *HIV* Human Immunodeficiency Virus, *ICU* Intensive care Unit, *SAPS II* Simplified Acute Physiology Score II, *SOFA* Sequential Organ Failure Assessment

^a^Missing data: n = 29; ^b^Missing data: n = 1; ^c^Missing data: n = 2; ^d^Missing data: n = 26; e missing data: n = 20, ^f^Missing data: n = 6; ^g^Missing data: n = 9; ^h^Missing data: n = 10

### IPTW analysis of intubation risk

**Fig. 2:**
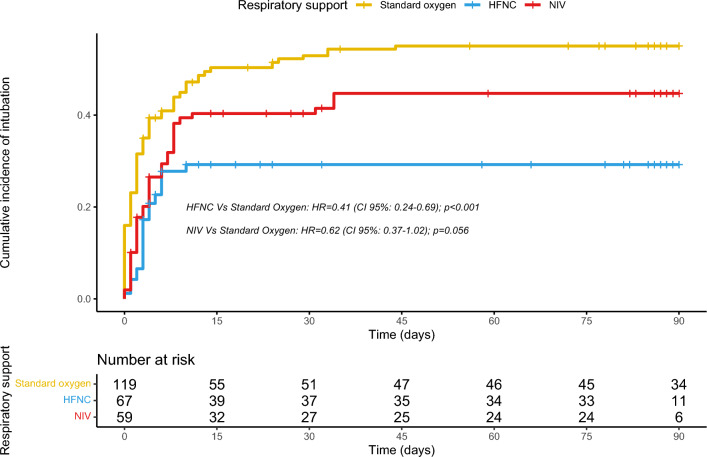
Weighted cumulative incidence of intubation in critically ill pneumocystis pneumonia patients according to respiratory management

In order to overcome baseline differences between groups and to take into account potential confounders, an IPTW analysis was performed. The baseline characteristics between the three groups were reassessed after IPTW. The standardized mean differences of each variable are shown in Supplementary Fig. 1. The baseline characteristics between the three groups after IPTW appeared well balanced (SMD < 0.15) (Supplementary Table 1). When assessing the cumulative incidence of intubation, through time dependent weighted Cox regression we observe that HFNC was associated with a lower likelihood of requiring intubation (Weighted HR 0.41 (95% CI 0.24–0.69); p < 0.001). Conversely, the potential protective effect of NIV was not evidenced (HR 0.62 (95% CI 0.37–1.02); p = 0.056). Incidence curves are displayed in Fig. 2.

### Survival analysis

Through adjusted survival analysis, long term corticosteroids treatment (aHR 4.03 (95% CI 2.01–8.08); p < 0.001), ST (aHR 3.37 (95% CI 1.45–7.86); p = 0.005) and the SOFA score (aHR 1.24 (95% CI 1.15–1.35); p < 0.001) were found to be independent predictor for death. Conversely, although by univariable analysis HFNC was associated with survival (HR 0.56 (95% CI 0.33–0.96); p = 0.036), adjusted analysis did not evidenced such a statistically significant result (aHR 0.91 (95% CI 0.46–1.77); p = 0.828) (Table 3). In addition, we conducting an IPTW analysis of survival with covariates associated with survival and ventilatory support. After IPTW, baselines characteristics appeared broadly balanced (Supplementary Fig. 2). Initial ventilatory support was not evidenced of being associated with survival (weighted HR 0.86 (95% CI 0.43–1.73); p = 0.668 for HFNC versus SO and weighted HR 0.72 (95% CI 0.35–1.51); p = 0.392 for NIV versus SO). Finally, by performing a sensitivity analysis of survival including mechanical ventilation, we observed that intubation was an independent predictor of death (aHR 2.63 (95% CI 1.39–4.97); p = 0.003) (Supplementary Table 2).

**Table 3:** Survival analysis of critically ill pneumocystis pneumonia patients

	Non adjusted analysis			Adjusted analysis		
	HR	95%CI	*p* value	aHR	95%CI	*p* Value
Baseline features						
Age (years)	10.02	1.01–1.04	0.002	1.01	0.99–1.03	0.332
Male sex	0.87	0.56–1.34	0.523			
Comorbidities						
Obesity (BMI > 30)	0.46	0.14–1.46	0.187	0.51	0.15–1.68	0.266
Chronic renal disease	10.33	0.87–2.03	0.188	0.60	0.35–1.05	0.075
Chronic pulmonary disease	10.24	0.76–2.00	0.389			
Long term corticosteroids treatment	20.02	1.29–3.16	0.002	4.03	2.01–8.08	< 0.001
Underlying cause of immunosuppression						
HIV	0.39	0.20–0.75	0.005	2.39	0.90–6.31	0.079
Solid organ transplantation	10.10	0.67–1.80	0.696			
Hematological malignancy	0.93	0.56–1.55	0.786			
Solid tumor	20.35	1.27–4.32	0.006	3.37	1.45–7.86	0.005
Immune-mediated inflammatory disease	10.35	0.83–2.17	0.222			
Initial laboratory findings and management						
Anti pneumocystis prophylaxis	0.76	0.36–1.56	0.451			
CRP (mg/L)	1.00	1.00–1.00	0.285			
PaO_2_ to F_i_O_2_ ratio	0.99	0.99–1.00	< 0.001	1.00	0.99–1.00	0.199
PaCO_2_ at ICU admission	1.00	0.99–1.02	0.675			
SAPS II	1.03	1.02–1.04	< 0.001	1.01	1.00–1.03	0.066
SOFA	1.26	1.21–1.33	< 0.001	1.24	1.15–1.35	< 0.001
Respiratory management						
Standard oxygen	ref	ref	ref	ref	ref	ref
NIV	0.61	0.35–1.06	0.079	0.93	0.49–1.76	0.773
HFNC	0.56	0.33–0.96	0.036	0.91	0.46–1.77	0.828

*BMI* Body mass index, *CRP* C-reactive Protein, *HFNC* High-Flow Nasal Cannula, *HIV* Human Immunodeficiency Virus, *SAPS II* Simplified Acute Physiology Score II

## Discussion

Through this large cohort study of critically ill PjP patients, HFNC was associated with a reduction in the need for MV compared with standard oxygen. Nevertheless, such a substantial reduction in mechanical ventilation did not translate into a higher survival rate.

Although ARF is the hallmark of PjP, respiratory strategies remain poorly explored. A recent randomized controlled trial (RCT) conducted in HIV patients with ARF (PjP being the most main etiology for ARF) comparing HFNC with non-invasive ventilation did not show any difference in cumulative intubation rates [18]. While in a multicenter cohort study dedicated to immunocompromised patients, HFNC appeared to have an effect on intubation but not on mortality rates [7]. Conversely, the HIGH RCT did not evidence any benefit of HFNC over SO [10]. Discrepancies across studies might have been favored by heterogeneity in both underlying causes of immunosuppression and causes of ARF.

The physiological effects of HFNC including less inspiratory effort and improved lung volume and compliance [19] could be particularly beneficial in PjP patients. Additionally, HFNC may reduce the risk of NIV-associated complications such as barotrauma due to elevated airway pressure along with potential exacerbation of the work of breathing in patients with poor pulmonary compliance, and the risk of delayed intubation in the context of progressive respiratory failure [20, 21].

Prophylaxis of PjP is the cornerstone of its prevention. As evidenced previously [3], the proportion of patients receiving such a prophylaxis is low in the present cohort of severe PjP patients. Moreover, since patients with ST and long-term corticosteroids appear to have the poorest prognosis, clinicians should be aware that prevention of PJP is particularly crucial in these patients.

In the present study, intubation appeared to represent a critical turning point, with a significant increase in mortality among PjP patients requiring MV (either by univariable analysis or through multivariable Cox regression). Although we did not evidence a significant impact on survival by multivariable analysis, our study was not designed for this purpose and the sample size of the present cohort might be too small to assess such a critical issue. In addition, as recently demonstrated, immunocompromised patients (and per se, critically ill PjP patients) represent a highly heterogenous group with various phenotypes (and different severity notably depending on underlying cause of immunosuppression [2, 5, 22]) in which specific intervention (such as HFNC) are more likely to be beneficial [23]. Therefore, a more tailored approach might be needed in such a heterogenous population. Finally, through a reduction in the need of mechanical ventilation, HFNC might also promote a lower likelihood of developing ICU-acquired infections such as ventilator associated pneumonia and *Herpesviridae* reactivations (which were shown to be associated with worsen outcomes [11, 24]).

This study is, to our knowledge, the first one to assess the effects of HFNC compared to SO and NIV in PjP patients. Nonetheless some limitations must be acknowledged. The retrospective design of our study inherently carries a risk of residual confounding, limiting the the ability to draw definitive conclusions. Although we performed an IPTW analysis to adjust for observed covariates, unmeasured factors may have influenced clinicians' choices of ventilatory strategies, potentially introducing selection bias. A randomized controlled trial would be necessary to robustly assess the impact of different respiratory strategies in PjP patients. In addition, the several years between first and last inclusion as well as heterogeneity among participating centers in ARF strategies may be residual confounding factors that could explain the present findings. Additionally, due to the retrospective design of our study, time to the different respiratory supports might have varied across patient ‘groups and some patients could receive different respiratory supports which may limit the interpretation of the results. Finally, heterogeneity in underlying causes of immunosuppression may make definitive conclusion difficult to be drawn.

To conclude, although oxygenation strategy was not associated with D90 survival in critically ill PjP patients, HFNC support appeared to be associated with a lower intubation rate. Further prospective studies are warranted to refine respiratory management in critically ill PjP patients.

## Supplementary Information

Supplementary material 1.

Supplementary material 2.

## Data Availability

The datasets from this study are available from the corresponding author on request.

## Abbreviations

ARF: Acute respiratory failure
CI: Confidence interval
CRP: C-Reactive Protein
FIO2: Inspiratory oxygen fraction
HIV: Human immunodeficiency virus
HFNC: High-Flow Nasal Cannula
HM: Hematological malignancies
HR: Hazard Ratio
IMID: Immune-mediated inflammatory disease
ICU: Intensive care unit
IQR: Interquartile range
MV: Invasive mechanical ventilation
NIV: Non-invasive ventilation
OR: Odds ratio
PaO2: Arterial partial pressure of oxygen
PCR: Real-time polymerase chain reaction
PjP: Pneumocystis jirovecii pneumonia
SAPAII: Simplified Acute Physiological Score II
SD: Standard deviation
SO: Standard oxygen SO
SOFA score: Sequential Organ Failure Assessment score
SOT: Solid organ transplantation
