# Supplementary material for: Respiratory management of critically ill pneumocystis pneumonia patients: a multicenter retrospective study
Source: Ann Intensive Care. 2025 Aug 6;15:114. doi: 10.1186/s13613-025-01503-6 (PMC12328854; doi:10.1186/s13613-025-01503-6)
Supplement: Supplementary file 1 — Supplementary material 1. [file 13613_2025_1503_MOESM1_ESM.pptx]

## Slide 1
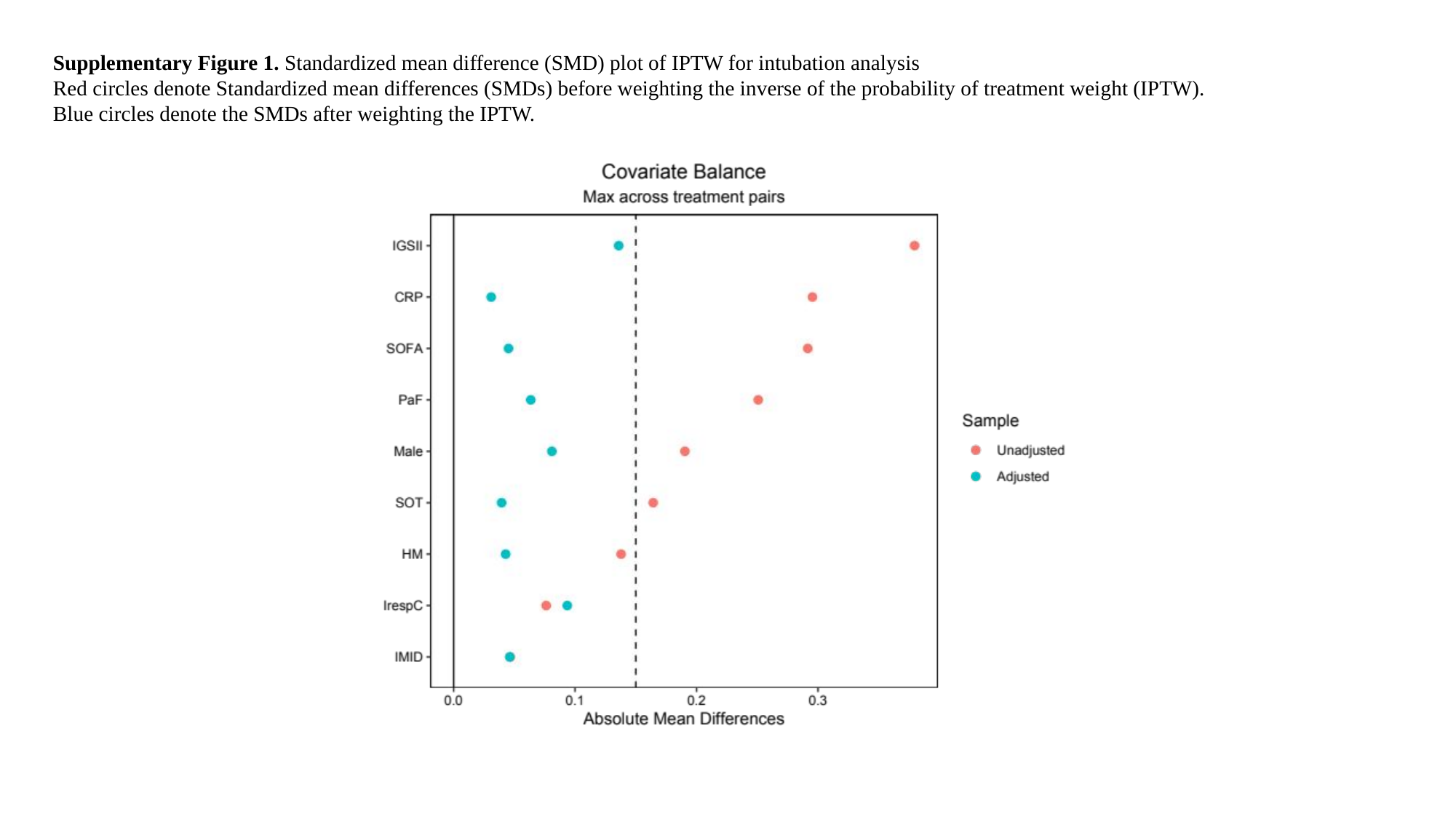

Supplementary Figure 1. Standardized mean difference (SMD) plot of IPTW for intubation analysis
Red circles denote Standardized mean differences (SMDs) before weighting the inverse of the probability of treatment weight (IPTW).
Blue circles denote the SMDs after weighting the IPTW.

## Slide 2
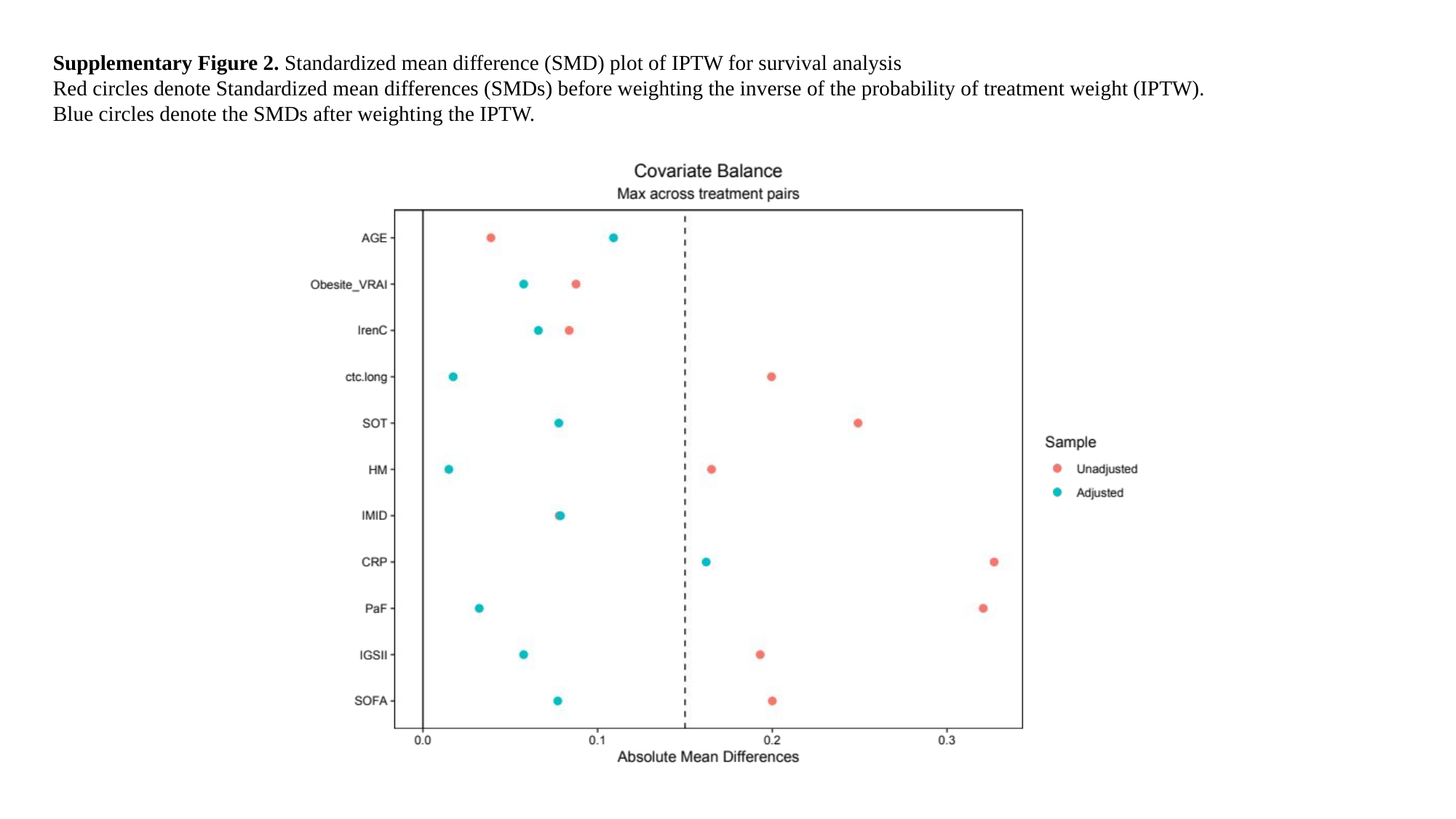

Supplementary Figure 2. Standardized mean difference (SMD) plot of IPTW for survival analysis
Red circles denote Standardized mean differences (SMDs) before weighting the inverse of the probability of treatment weight (IPTW).
Blue circles denote the SMDs after weighting the IPTW.

## Slide 3
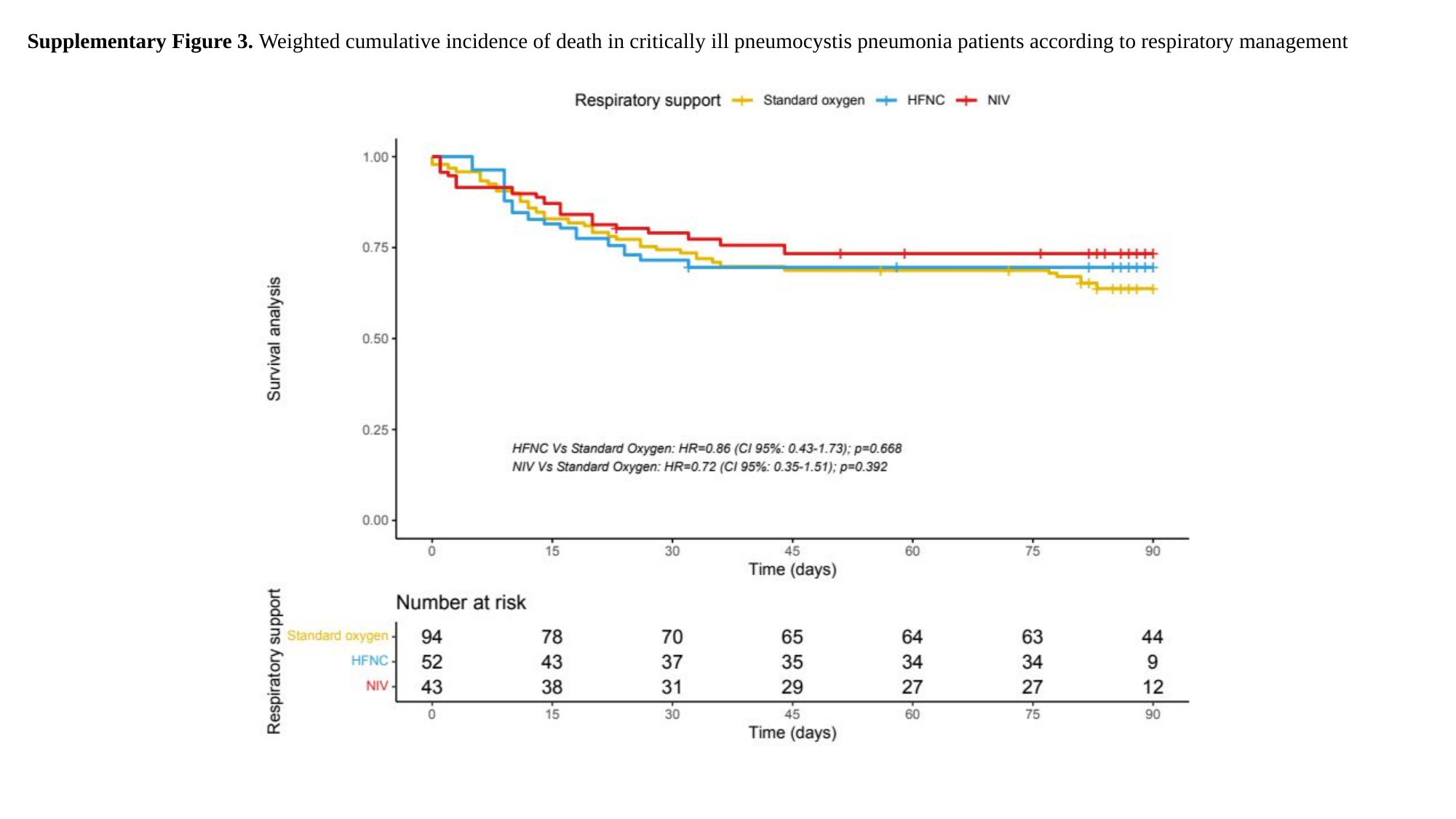

Supplementary Figure 3. Weighted cumulative incidence of death in critically ill pneumocystis pneumonia patients according to respiratory management
