# Supplementary material for: Respiratory management of critically ill pneumocystis pneumonia patients: a multicenter retrospective study
Source: Ann Intensive Care. 2025 Aug 6;15:114. doi: 10.1186/s13613-025-01503-6 (PMC12328854; doi:10.1186/s13613-025-01503-6)
Supplement: Supplementary file 2 — Supplementary material 2. [file 13613_2025_1503_MOESM2_ESM.docx]

**Supplementary Table 1.** Baseline and weighted covariates used for IPTW of critically ill pneumocystis pneumonia patients

|  |  | | **Unweighted** | | | | |  | | **Weighted** | | |
| --- | --- | --- | --- | --- | --- | --- | --- | --- | --- | --- | --- | --- |
|  | | Standard Oxygen  n=118 | | HFNC  n=70 | NIV  n=60 | SMD | Standard Oxygen  n=119 | | HFNC  n=66 | | NIV  n=59 | SMD |
| Male sex | | 68 (57.6) | | 41 (58.6) | 46 (76.7) | 0.190 | 75.0 (62.9) | | 41.0 (61.6) | | 40.8 (69.7) | 0.081 |
| Hematological malignancy | | 32 (27.1) | | 18 (25.7) | 8 (13.3) | 0.138 | 27.8 (23.3) | | 15.0 (22.5) | | 15.7 (26.8) | 0.043 |
| Solid organ transplantation | | 22 (18.6) | | 13 (18.6) | 21 (35.0) | 0.164 | 26.1 (21.9) | | 12.1 (18.2) | | 13.0 (22.1) | 0.039 |
| IMID | | 24 (20.3) | | 15 (21.4) | 15 (25.0) | 0.047 | 27.1 (22.7) | | 16.2 (24.4) | | 11.6 (19.8) | 0.046 |
| CRP | | 95 [49-181] | | 131 [60-198] | 124 [67-188] | 0.295 | 103 [49-184] | | 122 [55-196] | | 99 [29-200] | 0.031 |
| PaO_2_ to F_i_O_2_ ratio | | 135 [92-215] | | 122 [90-177] | 155 [94-220] | 0.251 | 131 [88-209] | | 145 [90-202] | | 155 [96-202] | 0.063 |
| SAPS II | | 39 [29-49] | | 37 [30-41] | 39 [31-50] | 0.379 | 37 [27-47] | | 38 [30-44] | | 37 [29-47] | 0.136 |
| SOFA | | 4 [3-7] | | 3 [3-5] | 4 [3-5] | 0.045 | 4 [3-6] | | 4 [3-6] | | 4 [3-6] | 0.045 |

Data are presented as median (IQR: interquartile)-n (%).

Abbreviations: CRP: C-Reactive Protein; SMD: Standardized Mean Difference; IMID: Immune-mediated inflammatory disease; SOFA: Sequential Organ Failure Assessment

**Supplementary Table 2.** Survival analysis of critically ill pneumocystis pneumonia patients (including mechanical ventilation)

|  | **Non adjusted analysis** | | |  | **Adjusted analysis** | | |  |
| --- | --- | --- | --- | --- | --- | --- | --- | --- |
|  | **HR** | **95%CI** | ***p* value** |  | **aHR** | **95%CI** | ***p* Value** |  |
| **Baseline features** |  |  |  |  |  |  |  |  |
| Age (years) | 1.02 | 1.01-1.04 | 0.002 |  | 1.02 | 0.99-1.04 | 0.164 |  |
| Male sex | 0.87 | 0.56-1.34 | 0.523 |  |  |  |  |  |
| **Comorbidities** |  |  |  |  |  |  |  |  |
| Obesity (BMI>30) | 0.46 | 0.14-1.46 | 0.187 |  | 0.61 | 0.19-1.97 | 0.404 |  |
| Chronic renal disease | 1.33 | 0.87-2.03 | 0.188 |  | 0.63 | 0.35-1.09 | 0.098 |  |
| Chronic pulmonary disease | 1.24 | 0.76-2.00 | 0.389 |  |  |  |  |  |
| Long term corticosteroids treatment | 2.02 | 1.29-3.16 | 0.002 |  | 4.52 | 2.22-9.20 | <0.001 |  |
| **Underlying cause of immunosuppression** |  |  |  |  |  |  |  |  |
| HIV | 0.39 | 0.20-0.75 | 0.005 |  | 2.41 | 0.89-6.51 | 0.084 |  |
| Solid organ transplantation | 1.10 | 0.67-1.80 | 0.696 |  |  |  |  |  |
| Hematological malignancy | 0.93 | 0.56-1.55 | 0.786 |  |  |  |  |  |
| Solid tumor | 2.35 | 1.27-4.32 | 0.006 |  | 4.85 | 2.06-11.43 | <0.001 |  |
| Immune-mediated inflammatory disease | 1.35 | 0.83-2.17 | 0.222 |  |  |  |  |  |
| **Initial laboratory findings and management** |  |  |  |  |  |  |  |  |
| Anti pneumocystis prophylaxis | 0.76 | 0.36-1.56 | 0.451 |  |  |  |  |  |
| CRP (mg/L) | 1.00 | 1.00-1.00 | 0.285 |  |  |  |  |  |
| PaO_2_ to F_i_O_2_ ratio | 0.99 | 0.99-1.00 | <0.001 |  | 1.00 | 1.00-1.00 | 0.961 |  |
| PaCO_2_ at ICU admission | 1.00 | 0.99-1.02 | 0.675 |  |  |  |  |  |
| SAPS II | 1.03 | 1.02-1.04 | <0.001 |  | 1.02 | 1.00-1.03 | 0.025 |  |
| SOFA | 1.26 | 1.21-1.33 | <0.001 |  | 1.24 | 1.1-1.35 | <0.001 |  |
| Mechanical ventilation | 3.81 | 2.39-6.08 | <0.001 |  | 2.63 | 1.39-4.97 | 0.003 |  |

Abbreviations: BMI: Body mass index; CRP: C-reactive Protein; HFNC: High-Flow Nasal Cannula; HIV: Human Immunodeficiency Virus; SAPS II: Simplified Acute Physiology Score II
